# Effects of Mexiletine on a Race-specific Mutation in Na_v_1.5 Associated With Long QT Syndrome

**DOI:** 10.3389/fphys.2022.904664

**Published:** 2022-07-05

**Authors:** Xin Wu, Yawei Li, Liang Hong

**Affiliations:** ^1^ Department of Medicine, University of Illinois at Chicago, Chicago, IL, United States; ^2^ Department of Preventive Medicine, Northwestern University, Chicago, IL, United States

**Keywords:** voltage-gated sodium channel, Nav1.5, LQT syndrome, electrophysiology, patch clamp

## Abstract

The voltage-gated sodium channel Na_v_1.5 plays an essential role in the generation and propagation of action potential in cardiomyocytes. Mutations in Na_v_1.5 have been associated with LQT syndrome, Brugada syndrome, and sudden arrhythmia death syndrome. Genetic studies showed that Na_v_1.5 mutations vary across race-ethnic groups. Here we investigated an Asian-specific mutation Na_v_1.5-P1090L associated with LQT syndrome. We found that Na_v_1.5-P1090L mutation perturbed the sodium channel function. It altered the gating process of the channel and exhibited an enhanced window current. Treatment with mexiletine reversed the depolarization shift of the steady-state inactivation produced by P1090L. Mexiletine also modified the recovery from steady-state inactivation and the development of inactivation of P1090L. It rescued the dysfunctional inactivation of P1090L and reduced the P1090L channel’s availability.

## Introduction

Long QT syndrome (LQTS) is a heart rhythm disorder and increases risk for life-threatening sudden cardiac arrest (Napolitano et al., 2005; Ware et al., 2012). It is an inherited arrhythmia associated with ion channel mutations (Grant et al., 2002; Kapa et al., 2009; Chiu et al., 2012; Wilde and Amin, 2018).

The voltage-gated sodium channel Na_v_1.5, encoded by SCN5A, plays a critical role in the fast depolarization of the cardiac action potential duration (APD) (Dong et al., 2020; Wu and Hong, 2021). Genetic mutations in Na_v_1.5 linked with long QT syndrome type 3 (LQTS3) affected channel’s function and remodeled ventricular APD (Bankston et al., 2007; Wilde and Amin, 2018). Some Na_v_1.5 mutations have been shown to be race-specific. It was reported that Na_v_1.5 mutations R34C and S1103Y were common variants in black cohort; and R1193Q and P1090L were observed predominantly among Asian patients (Ackerman et al., 2004). Although epidemiological research provided important insights into Na_v_1.5 mutations, the role of some race-specific mutations in LQTS remained unclear.

Here we investigated an Asian-specific mutation Na_v_1.5-P1090L associated with LQTS. The Na_v_1.5 mutation P1090L was first reported to be linked with familial long QT syndrome in Japanese population (Iwasa et al., 2000). It was identified as an ethnic-specific variant in Asians (Ackerman et al., 2004). The Na_v_1.5-P1090L has also been associated with Brugada syndrome, sudden infant death syndrome, sick sinus syndrome, and other severe arrhythmia (Ackerman et al., 2004; Lai et al., 2005; Tan et al., 2005; Shan et al., 2008; Zhang et al., 2008; Nakajima et al., 2011; Chiu et al., 2012) (Table 1). To explore the role of the P1090L in the LQTS, we characterized effects of the mutation on the channel function. We showed that P1090L perturbed the gating process of the sodium channel. It generated a larger window current associated with a gain-of-function mechanism underlying LQTS. We then studied effects of a targeted drug on the Na_v_1.5-P1090L mutation.

**TABLE 1:** Summary of citations for the Na_v_1.5 mutation P1090L associated with cardiac arrhythmias.

Nav1.5 Variant	Change in Nucleotide	Phenotype	References
P1090L	c. 3269C > T	Long QT syndrome	Iwasa et al. (2000)
		Brugada/Long QT syndrome	Ackerman et al. (2004)
		Arrhythmia	Tan et al. (2005)
		Long QT syndrome	Lai et al. (2005)
		Premature ventricular contraction/Sick sinus syndrome	Shan et al. (2008)
		Arrhythmia	Zhang et al. (2008)
		Brugada syndrome	Nakajima et al. (2011)
		Long QT syndrome	Chiu et al. (2012)

## Materials and Methods

### Cell Culture and Na_v_1.5 Transfection

HEK-293T cell lines (Sigma-Aldrich, St. Louis, United States) were used to express human Na_v_1.5 wild-type (WT) and mutation P1090L. The cells were reseeded on coverslips in 6-well plates containing Dulbecco modified Eagle’s medium (DMEM, Invitrogen) supplemented with 10% fetal bovine serum (Invitrogen), 100 U/mL penicillin, and 100 μg/ml streptomycin at 37°C under 5% CO_2_. After growth to ∼80% confluence, cells were transiently transfected with Na_v_1.5 cDNA plasmids. The pRc/CMV plasmid containing the sequence of the human Na_v_1.5 channel was kindly provided by Dr Alfred L. George Jr (Northwestern University, Chicago, IL, United States). Single-point mutation of P1090L in pRc/CMV-hNa_v_1.5 was introduced with standard PCR techniques. The mutation was introduced to the template plasmid using primers in a PCR protocol. The PCR cycles were initiated at 98°C for 1.5 min, followed by 25 amplification cycles. Each amplification cycle consisted of 98°C (20 s), 60°C (30 s), and 72°C (5 min). The cycles were finished with an extension step at 72°C for 15 min, followed by 4°C for 30 min. The template plasmid was removed using DpnI (NEB, R0176S), and Stbl2 competent cells (Invitrogen, 10268019) were transformed with the PCR product. Plasmids were isolated from Stbl2 cells with resulting colonies using QIAprep Spin Miniprep Kit (Qiagen, 27106). The mutation confirmed by the DNA sequencing was used for subsequent transfection.

The HEK-293T cells were transiently transfected with 2 μg of the cDNA encoding Na_v_1.5 WT or P1090L mutation and 0.25 μg of a plasmid encoding enhanced green fluorescent protein (GFP) using Lipofectamine 3000 reagent (Invitrogen; Thermo Fisher Scientific, Inc., United States) according to the manufacturer’s protocol. The mixture was then added to the culture dish and the cells were incubated at 37°C for 24 h before the electrophysiology studies were conducted. A coverslip with HEK-293T cells was placed in a recording chamber containing bath solution on the stage of a fluorescence microscope (Olympus, Japan), and the transfected cells identified by the fluorescent signal emitted from GFP were used for electrophysiological measurements. Patch clamp experiments were conducted 24–48 h after transfection.

### Electrophysiological Measurements and Analysis

Patch clamp measurements were performed in whole-cell configuration using an Axopatch 200B amplifier controlled by pClamp11 software through an Axon Digidata 1550B system (Molecular Devices, United States). The extracellular solution contained 130 mM NaCl, 5 mM KCl, 1 mM MgCl_2_, 2 mM CaCl_2_, 10 mM HEPES, adjusted to pH 7.4 with NaOH. The intracellular solution contained 120 mM CsF, 15 mM CsCl, 20 mM NaCl, 2 mM EGTA and 5 mM HEPES, adjusted to pH 7.3 with CsOH. All measurements were performed at 22 ± 3°C. Pipettes had 2–4 MΩ access resistance.

The activation G-V curves were fitted by the Boltzmann equation, as previously explained (Kim et al., 2014; Hong et al., 2020): G/G_max_ = 1/(1 + exp(V-V_1/2_)/k), where G/G_max_ is the relative conductance normalized by the maximal conductance, V_1/2_ is the potential of half activation, V is test pulse, and k is the Boltzmann coefficient. Steady-state inactivation curves were fitted by the Boltzmann equation: I/I_max_ = 1/(1 + exp(V-V_1/2_)/k), where I/I_max_ is the relative conductance normalized by the maximal conductance, V_1/2_ is half-maximal inactivation, V is test pulse, and k is the Boltzmann coefficient.

Exponential functions of the form y = y0+A(1-exp[-t/τ]) were fitted to development curves to determine time constant (Ʈ_dev_), where y0 is the offset and A is amplitude. The double exponential functions of the form y = A1(1-exp[-t/τ1])+A2(1-exp[-t/τ2]) were fitted to recovery curves to determine time constants (Ʈ_rec,f_ and Ʈ_rec,s_), A1 and A2 are the fractions of fast and slow inactivating components, and τ1 and τ2 are their time constants Ʈ_rec,f_ and Ʈ_rec,s_ respectively.

Dose-responses of channel inhibition by mexiletine were fitted by the Hill equation, as previously described (Zhao et al., 2021a; Zhao et al., 2021b): %_i_ = %_i,max_[B]^h^/(IC_50_^h^+[B]^h^), where %_i,max_ is the maximal percentage of channel inhibition by blocker B, h is the Hill coefficient, and IC_50_ is the concentration of mexiletine required for 50% inhibition.

### Data and Statistical Analysis

All data were presented as the mean ± SEM. Significance between means was determined by Student’s t-test. Electrophysiological parameters (V_1/2_, Ʈ_dev_, Ʈ_rec,f_, Ʈ_rec,s_) were determined from each individual cell and used for comparison with *t*-test. We repeated transfection experiments 3 or 4 times for each group cell, and 2 to 3 sodium currents were recorded in each independent culture. *p* < 0.05 was considered to indicate a statistically significant difference.

## Results

### Effects of P1090L on Na_v_1.5 Channel Function

**FIGURE 1:**
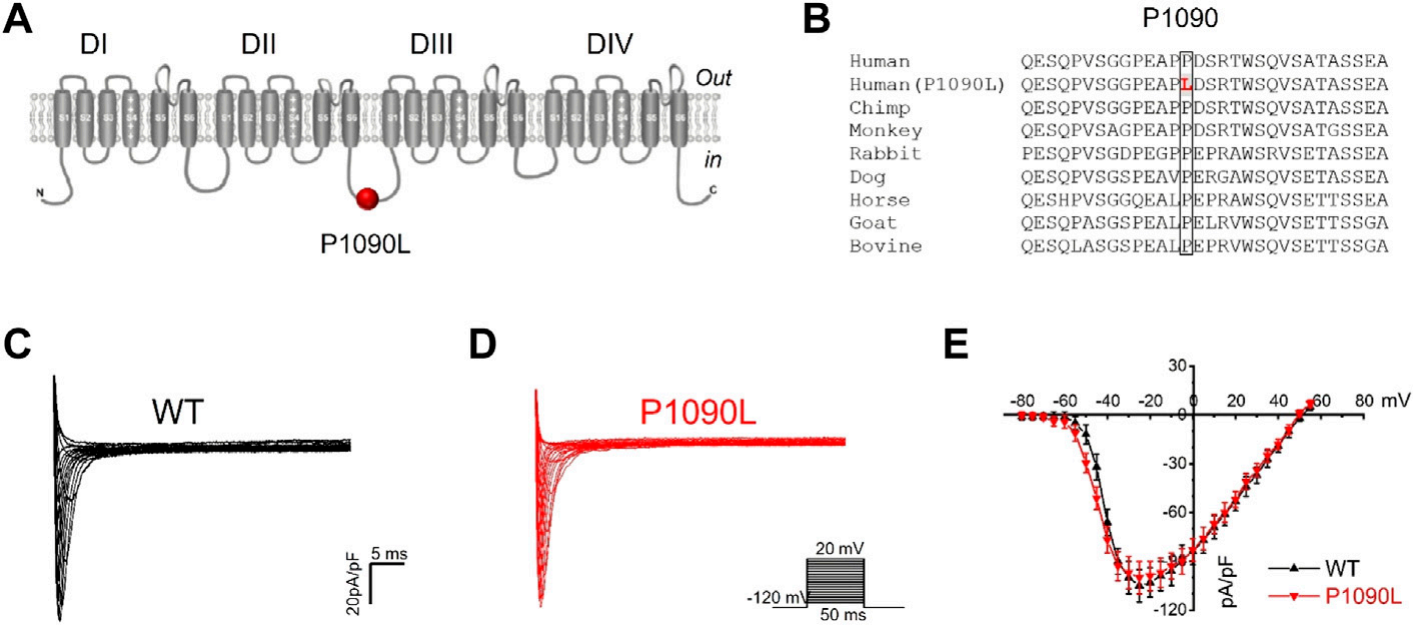
Asian-specific mutation P1090L in the Na_v_1.5 sodium channel. **(A)**. Topological diagram showing P1090L located in the intracellular loop between the DII-DIII domain of the Na_v_1.5 channel. **(B)**. Na_v_1.5 sequence alignment indicates that the P1090 is highly conserved across vertebrate species. **(C,D)**: Representative wild-type (WT) **(C)** and P1090L **(D)** sodium currents. Currents were measured from a holding potential of −120 mV to test potentials ranging between −110 and +60 mV in 5 mV steps. **(E)**. Average peak current-voltage (I-V) relationships for WT channel and P1090L mutation (*n* = 8 cells in each group, data are presented as the mean ± SEM).

The P1090 is located in the intracellular loop between the DII-DIII domain of the Na_v_1.5 channel (Figure 1A). Na_v_1.5 sequence alignment analysis shows that the native proline (P) at position 1090 is highly conserved across vertebrate species (Figure 1B).

**FIGURE 2:**
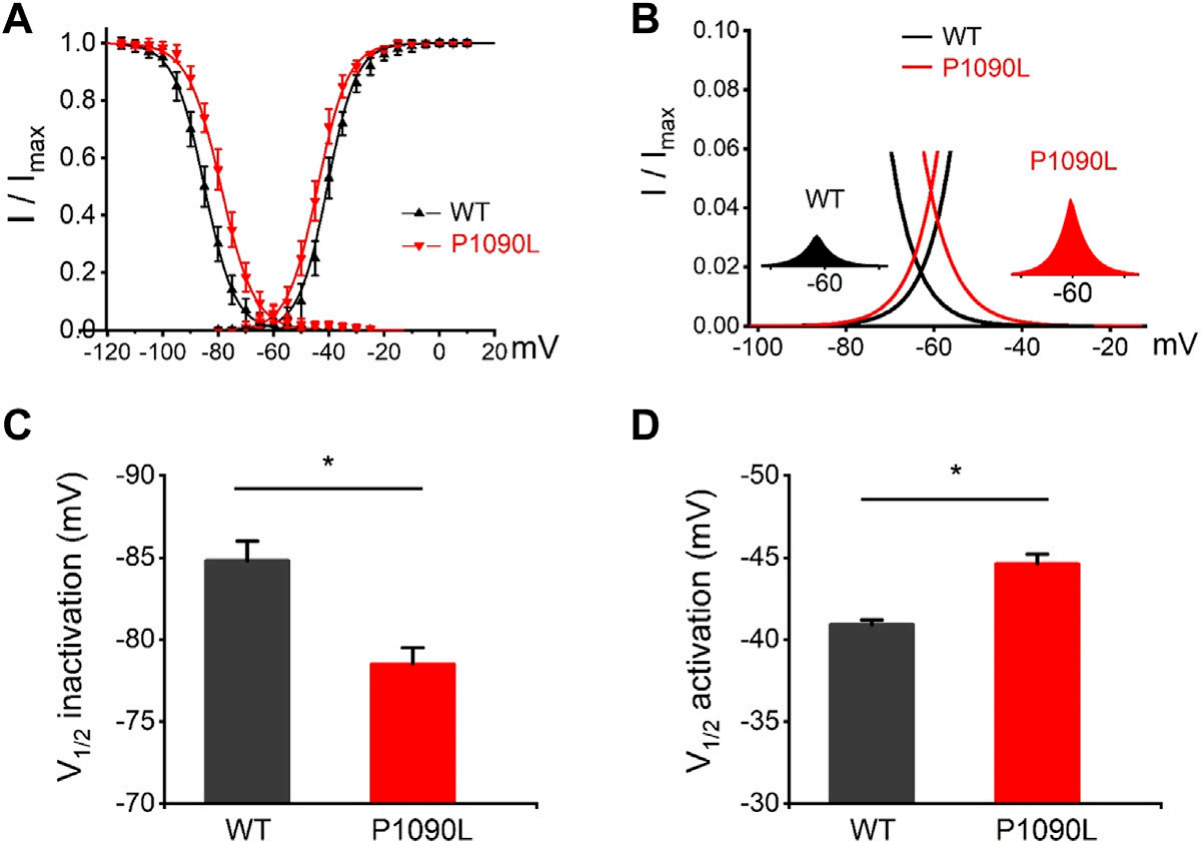
Effects of P1090L mutation on voltage-dependent gating of the Na_v_1.5 channel. **(A)**. Steady-state activation and inactivation curves of Na_v_1.5 WT and P1090L mutation. Inactivation currents were elicited to −20 mV following prepulses between −120 and −20 mV in 5 mV steps for 500 ms. Activation currents were measured from a holding potential of −120 mV to test potentials ranging between −110 and +20 mV in 5 mV steps. Curves are Boltzmann fits of the data points (*n* = 8). **(B)**. The view of the overlapping area between activation and steady-state inactivation was expanded from **(A)**. Left inset shows sodium window current of WT Na_v_1.5 channel (black). Right inset shows sodium window current of P1090L mutation (red). **(C)**. Summary of V_1/2_ of steady-state inactivation for WT and P1090L channel. **(D)**. Summary of V_1/2_ of voltage-dependent activation for WT and P1090L channel. Data are presented as the mean ± SEM. *p < 0.05.

Using whole cell patch clamp configuration, we characterized the biophysical properties of the P1090L mutation. The Na_v_1.5 wild-type (WT) and P1090L mutation in transiently transfected HEK293T cells were studied. We did not observe that P1090L altered the current amplitudes compared with WT (Figures 1C–E). However, P1090L shifted voltage dependence of activation to hyperpolarized direction (−44.6 ± 0.6 mV *vs*. −40.9 ± 0.3 mV, *n* = 8), and shifted voltage dependence of steady-state inactivation to depolarized direction (−78.5 ± 1.0 mV *vs*. −84.8 ± 1.2 mV, *n* = 8) (Figure 2). The depolarizing shift in steady-state inactivation and hyperpolarizing shift in activation in P1090L channel produced an overlap of activation and inactivation curves, resulting in a larger ‘‘window current” compared with WT channel (Figure 2B). This larger window current indicated a gain-of-function mechanism with an increase in sodium channel availability, which might enhance the excitability of myocytes and induce a prolonged ventricular APD underlying LQTS (Wilde and Amin, 2018).

### Effects of P1090L on the Channel Inactivation

**FIGURE 3:**
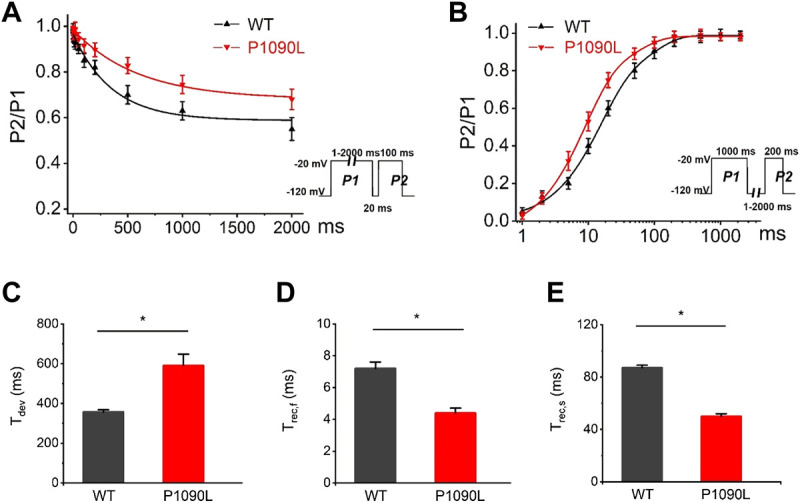
Effects of P1090L mutation on the channel inactivation. **(A)**. Development of inactivation for WT and P1090L mutation was assessed by varying the duration of a conditioning pulse P1 (1–2000 ms) to −20 mV followed by a return to -120 mV for 20 ms followed by a 100 ms test pulse P2 to −20 mV. **(B)**. Recovery of inactivation for WT and P1090L mutation was assessed using insert protocol with a first pulse duration of 1000 ms (P1) and a second pulse of 200 ms (P2) to −20 mV with varying rest intervals from 1 to 2000 ms at −120 mV. Curves in **(A,B)** are exponential function fits of the data points, *n* = 8 in each group. **(C)**. Summary of time constant for development (Ʈ_dev_) for WT and P1090L channel. **(D)**. Summary of time constant for fast recovery (Ʈ_rec,f_) for WT and P1090L. **(E)**. Summary of time constant for slow recovery (Ʈ_rec,s_) for WT and P1090L. Data are presented as the mean ± SEM. *p < 0.05.

**FIGURE 4:**
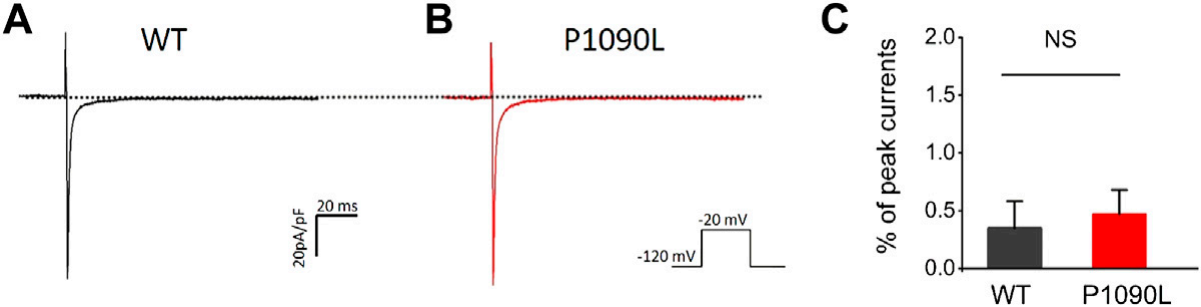
P1090L mutation does not increase the late sodium currents of the Na_v_1.5 channel. **(A,B)**: Representative wild-type (WT) **(A)** and P1090L **(B)** persistent sodium current traces. Currents were activated by depolarization to −20 mV from a holding potential of −120 mV, and the dash line represented 0 pA. **(C)**. The late sodium currents were measured and normalized to peak currents, *n* = 6 cells in each group, data are presented as the mean ± SEM. NS, non-significant.

To determine the effects of P1090L mutation on the process of the inactivation, we assessed the development and recovery of inactivation. The results showed that P1090L increased the time constant for development (Ʈ_dev_) (591 ± 27 ms *vs.* 357 ± 11 ms, *n* = 8), and decreased fast recovery time constant (Ʈ_rec,f_) (4.4 ± 0.3 ms *vs*. 7.2 ± 0.4 ms, *n* = 8) and slow recovery time constant (Ʈ_rec,s_) (50 ± 2 ms *vs*. 87 ± 2 ms, *n* = 8) (Figure 3), suggesting that the mutation delayed the process of development and enhanced the recovery from inactivated states of the channel. We also investigated the late sodium current, and P1090L mutation did not show larger persistent inward sodium current compared with WT channel (Figure 4).

### Mexiletine Inhibited P1090L Current

Since P1090L exhibited an increased window current, we wondered whether we could identify antiarrhythmic drug eliminating the increased window current. The mexiletine was reported to target Na_v_1.5 mutations and modulate sodium channel function (Ruan et al., 2007; Wang et al., 2015), and it could induce Na_v_1.5 mutated channels to enter an inactive state to enhance steady-state inactivation process, and decrease channel availability (Sasaki et al., 2004).

**FIGURE 5:**
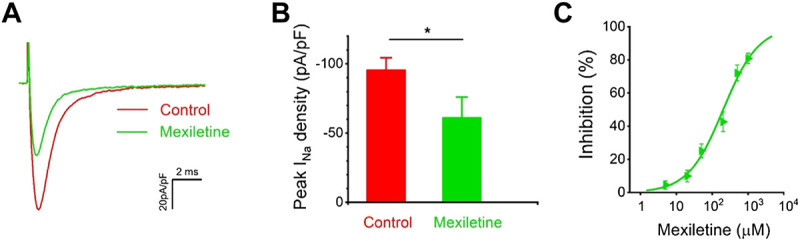
Inhibition of P1090L mutation currents by mexiletine. **(A)**. Representative current traces of P1090L recorded in the absence (Control) and presence of 200 µM mexiletine. Currents were elicited by a test pulse of −20 mV from a holding potential of −120 mV. **(B)**. Quantification of P1090L mutation current densities without (Control) or with the treatment of 200 µM mexiletine. *n* = 6 for each group, data are presented as the mean ± SEM. *p < 0.05. **(C)**. Dose-dependent effects of mexiletine on P1090L sodium current inhibition (*n* = 4-6 for each concentration; data are presented as the mean ± SEM). Curves are Hill fits of the data points.

We tested the effects of mexiletine on the P1090L peak sodium currents. The P1090L currents were measured prior to and after the addition of the mexiletine, and P1090L mutation currents were significantly inhibited by mexiletine (Figures 5A,B). Various concentrations of the drugs were used to generate a dose-response curve, and the IC_50_ value for mexiletine in P1090L mutation was 203 μM (Figure 5C). We next used 200 μM to study whether and how mexiletine affected the process of voltage-dependent inactivation in P1090L.

### Mexiletine Rescued the Dysfunctional Inactivation of the P1090L Mutation

**FIGURE 6:**
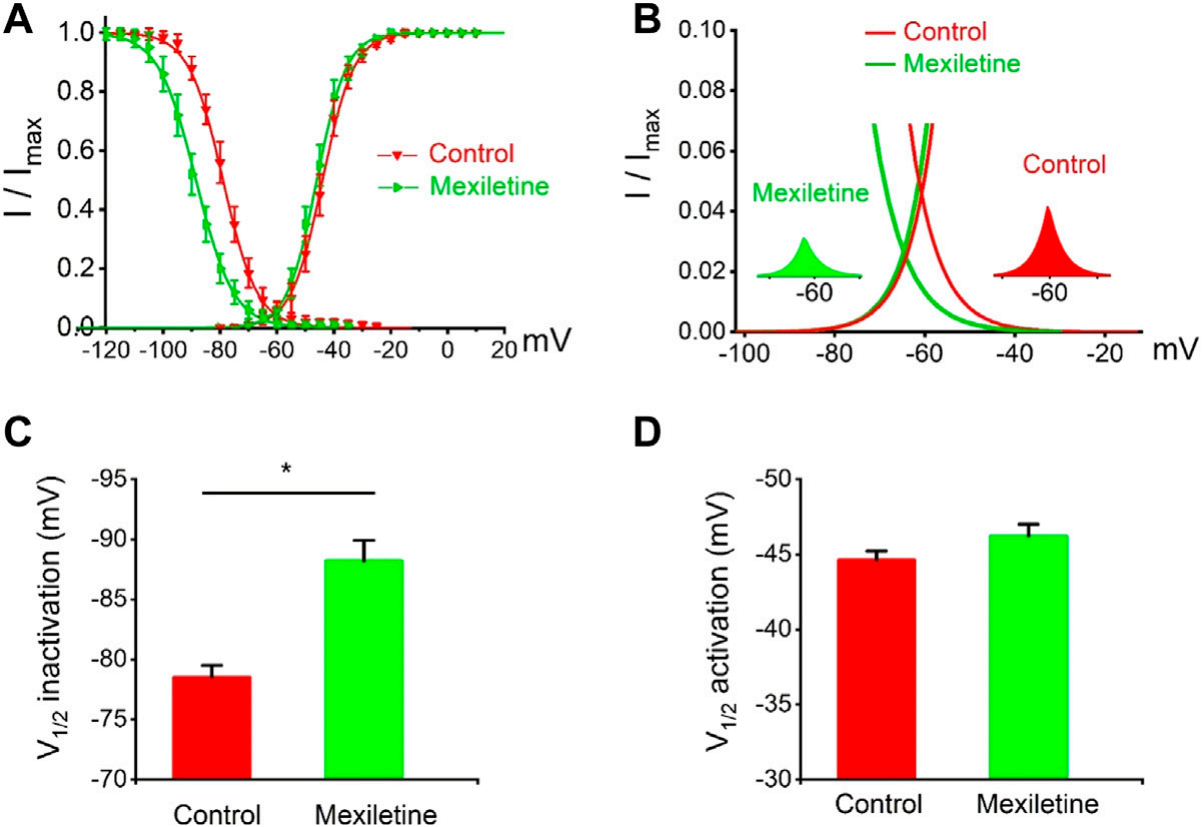
Effects of mexiletine on voltage-dependent gating of the P1090L mutation. **(A)**. Steady-state inactivation and activation curves of P1090L mutation currents in the absence (Control) and presence of 200 µM mexiletine. Inactivation currents were elicited to −20 mV following prepulses between −120 and −20 mV in 5 mV steps for 500 ms. Activation currents were measured from a holding potential of −120 mV to test potentials ranging between −110 and +20 mV in 5 mV steps. Curves are Boltzmann fits of the data points (*n* = 8 for Control, *n* = 6 for Mexiletine; data are presented as the means ± SEM). **(B)**. The view of the overlapping area between activation and steady-state inactivation was expanded from **(A)**. Right inset shows P1090L mutation sodium window current without mexiletine (Control), left inset shows P1090L window current in presence of the drug (Mexiletine). **(C)**. Summary of V_1/2_ of steady-state inactivation for control group and mexiletine group. **(D)**. Summary of V_1/2_ of voltage-dependent activation for control and mexiletine. Data are presented as the mean ± SEM. *p < 0.05.

**FIGURE 7:**
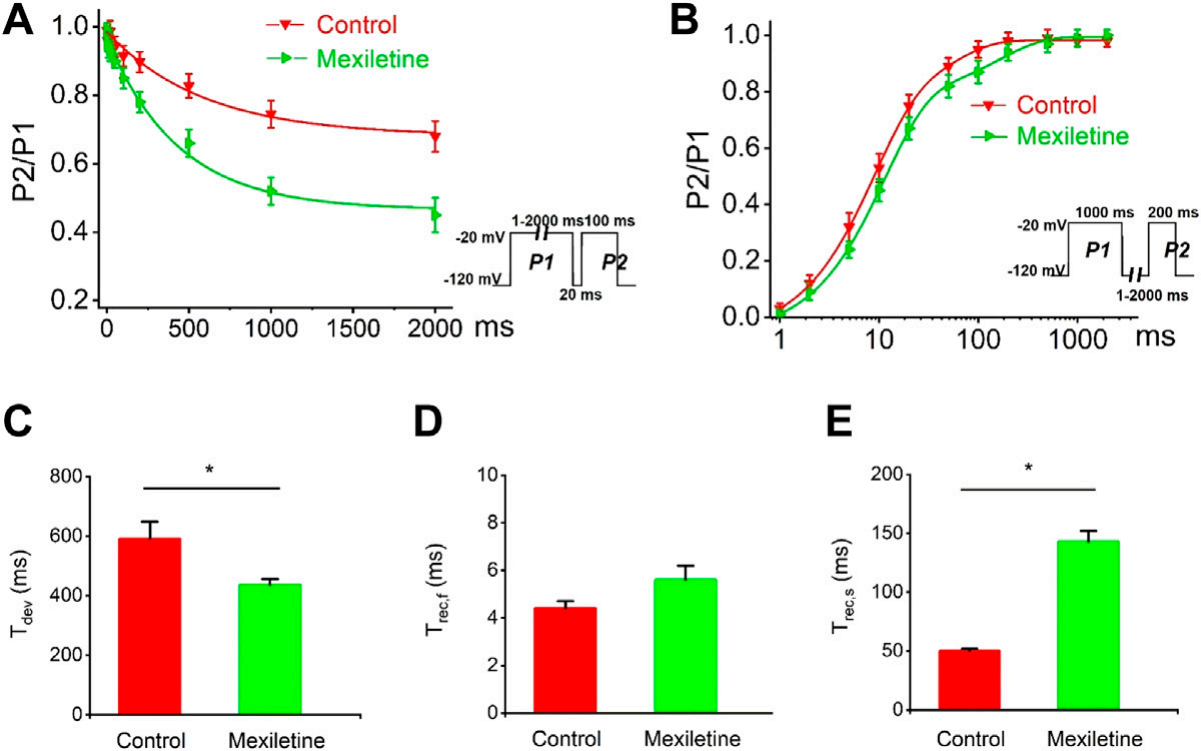
Effects of mexiletine on the development and recovery of inactivation of the P1090L mutation. **(A)**. Development of inactivation for P1090L mutation in the absence (Control) or presence of 200 µM mexiletine was assessed by varying the duration of a conditioning pulse P1 (1–2000 ms) to −20 mV followed by a return to −120 mV for 20 ms followed by a 100 ms test pulse P2 to −20 mV. **(B)**. Recovery of inactivation for P1090L mutation in the presence or absence of mexiletine was assessed using insert protocol with a first pulse duration of 1000 ms (P1) and a second pulse of 200 ms (P2) to −20 mV with varying rest intervals from 1 to 2000 ms at −120 mV. Curves in **(A,B)** are exponential function fits of the data points, *n* = 8 for Control, *n* = 6 for Mexiletine; data are presented as the mean ± SEM. **(C).** Summary of time constant for development (Ʈ_dev_) for control group and mexiletine group. **(D)**. Summary of time constant for fast recovery (Ʈ_rec,f_) for control and mexiletine. **(E)**. Summary of time constant for slow recovery (Ʈ_rec,s_) for control and mexiletine. Data are presented as the mean ± SEM. *p < 0.05.

We observed that the treatment with mexiletine shifted the steady-state inactivation of P1090L currents by around 10 mV to hyperpolarizing direction (−88.2 ± 1.7 mV *vs*. −78.5 ± 1.0 mV, *n* = 8 for control group, *n* = 6 for mexiletine group) with little effect on the channel activation (−46.2 ± 0.8 mV *vs*. −44.6 ± 0.6 mV, *n* = 8 for control, *n* = 6 for mexiletine) (Figure 6). The larger window current mediated by P1090L, obtained by plotting the area where steady-state inactivation and activation overlap, was notably reduced by mexiletine (Figure 6B). In addition, mexiletine rescued the abnormal inactivation process of the mutation (Figure 7). The drug modified development and recovery of inactivation in the mutated channel, and reduced Ʈ_dev_ (436 ± 20 ms *vs*. 591 ± 27 ms, *n* = 8 for control, *n* = 6 for mexiletine), and increased Ʈ_rec,s_ of P1090L (143 ± 9 ms *vs*. 50 ± 2 ms, *n* = 8 for control, *n* = 6 for mexiletine) (Figures 7C,E). These results indicated that mexiletine enhanced P1090L channel inactivation, stabilized the inactivation gate of the P1090L, and reduced the mutated channel availability.

## Discussion

In the present study, we showed that Na_v_1.5 mutation P1090L linked with LQTS perturbed the process of steady-state inactivation of the channel and generated an enhanced window current that is associated with a gain-of-function mechanism. Treatment with mexiletine rescued the dysfunctional inactivation of the mutation, and decreased the larger window sodium current mediated by P1090L.

The cardiac sodium channel Na_v_1.5 conducts inward sodium currents initiating the cardiac action potential (Dong et al., 2020). Mutations in Na_v_1.5 have been linked with inherited arrhythmias such as long QT syndrome (Lai et al., 2005), Brugada Syndrome (Nakajima et al., 2011), atrial fibrillation (Hong et al., 2021). There are two mechanisms proposed to reveal pathophysiological roles of Na_v_1.5 mutations in arrhythmias (Wilde and Amin, 2018). One is the loss-of function mechanism in which the mutation lacks the molecular function of the wild-type channel. The other is the gain-of-function mechanism in which the mutation changes gene product to confer new or enhanced activity underlying clinical phenotype. The gain-of-function mutations in the Na_v_1.5 channel normally generate an increase in the window current and/or the late sodium current, resulting in the prolongation of cardiac APD. Previous study reported a negative shift of activation in the Q1077del background for P1090L (Tan et al., 2005). Here we characterized that the P1090L is a gain-of-function mutation, and the mutation presented a larger window current and increased the channel availability. The window current is known as the ionic current flux through ion channels in a voltage range where steady-state inactivation and activation states overlap. The Na_v_1.5 channel window current usually controls action potential shape in myocytes. However, an enhanced window current may result in severe arrhythmia and heart failure.

Mexiletine is a voltage-gated sodium channel blocker, and the binding sites of mexiletine were proposed to be located at the intracellular side of the channel (Li et al., 2020). Mexiletine belongs to class IB antiarrhythmic agents, previous studies reported that mexiletine exerted a direct effect on LQTS mutations and modified Na_v_1.5 mutation currents (Kim et al., 2019; Li et al., 2020). A recent study reported differential gating properties of wild-type Na_v_1.5 and Na_v_1.7 channels in response to mexiletine and another drug (Wang et al., 2015). Mexiletine did not affect the voltage-dependent activation of the wild-type Na_v_1.5 channel; however, the steady-state inactivation of the wild-type Na_v_1.5 was significantly shifted to hyperpolarized direction by mexiletine in dose-dependent manner (Wang et al., 2015). In the present study, we characterized the effects of mexiletine on the mutated channel Na_v_1.5-P1090L, and showed mexiletine shifted the steady-state inactivation curve of P1090L to a hyperpolarizing direction, the drug rescued an abnormal development and recovery process of the inactivation state in the P1090L mutation. Our results are consistent with previous findings that mexiletine regulated the steady-state inactivation process and induced Na_v_1.5 mutated channel to enter an inactive state (Sasaki et al., 2004).

In LQTS patients carrying Nav1.5 gain-of-function mutations, an enhanced window current can increase the sodium ion entry into the cells during ventricular diastole. As a result, sodium currents are more expressed when plateau and repolarization phases last longer, which might explain the clinically observed slow-rate dependent QT prolongation. The larger sodium window current can promote delayed afterdepolarizations, or prolong cardiac action potential inducing the occurrence of early afterdepolarizations. Therefore, a targeted antiarrhythmic drug (AAD) that eliminates the larger window current would reduce the occurrence of arrhythmic events in patients. In the present study, we found that mexiletine reduced the larger window current mediated by the Nav1.5-P1090L mutation. Mechanism-based inhibition of the larger window current by a therapeutic drug would be expected to decrease arrhythmic events in Nav1.5-P1090L patients.

Recent studies on the Nav1.5-P1090L were focused on case reports or genetic analysis, and there was not yet a report on clinical applications of mexiletine in P1090L patients. Like the P1090L, another LQTS mutation Nav1.5-V411M also generates a larger sodium window current associated with the gain-of-function mechanism (Horne et al., 2011). The subsequent clinical investigation reported the consequences of therapy with mexiletine in Nav1.5-V411M patients. Mexiletine was shown to shorten the QT interval and efficaciously reduce arrhythmic events in LQTS patients (Mazzanti et al., 2016).

One limitation of the present study is that we used heterologous expression system to study the race-specific mutation Na_v_1.5-P1090L. As cardiomyocytes have distinct electrophysiological properties, the *in vitro* method could not capture full spectrum of functional changes of the LQTS-linked mutations. Meanwhile, studies showed that Na_v_1.5 mutation and other channel are regulators of gene transcriptional networks in cardiomyocytes (Hong et al., 2021; Wu et al., 2022), further studies in patient-specific iPSC-CMs (induced pluripotent stem cell derived cardiomyocytes) will help to explore cellular mechanism of the mutation (Itzhaki et al., 2011).

In summary, we characterized electrophysiological properties of Na_v_1.5 mutation P1090L linked with LQTS, and showed that mexiletine effectively reduced the enhanced window current generated by P1090L. The results provide insights into the mechanisms by which Na_v_1.5 mutation causes arrhythmias, and might potentially enable a mechanism-based approach to the treatment of LQTS.

## Data Availability

The original contributions presented in the study are included in the article, further inquiries can be directed to the corresponding author.
